# RetinaViT: Efficient Visual Backbone for Online Video Streams

**DOI:** 10.3390/s24175457

**Published:** 2024-08-23

**Authors:** Tomoyuki Suzuki, Yoshimitsu Aoki

**Affiliations:** Department of Electronics and Electrical Engineering, Faculty of Science and Technology, Keio University, 3-14-1, Hiyoshi, Kohoku-ku, Yokohama 223-8522, Kanagawa, Japan; aoki@elec.keio.ac.jp

**Keywords:** online video understanding, Vision Transformer, efficient computation

## Abstract

In online video understanding, which has a wide range of real-world applications, inference speed is crucial. Many approaches involve frame-level visual feature extraction, which often represents the biggest bottleneck. We propose RetinaViT, an efficient method for extracting frame-level visual features in an online video stream, aiming to fundamentally enhance the efficiency of online video understanding tasks. RetinaViT is composed of efficiently approximated Transformer blocks that only take changed tokens (event tokens) as queries and reuse the already processed tokens from the previous timestep for the others. Furthermore, we restrict keys and values to the spatial neighborhoods of event tokens to further improve efficiency. RetinaViT involves tuning multiple parameters, which we determine through a multi-step process. During model training, we randomly vary these parameters and then perform black-box optimization to maximize accuracy and efficiency on the pre-trained model. We conducted extensive experiments on various online video recognition tasks, including action recognition, pose estimation, and object segmentation, validating the effectiveness of each component in RetinaViT and demonstrating improvements in the speed/accuracy trade-off compared to baselines. In particular, for action recognition, RetinaViT built on ViT-B16 reduces inference time by approximately 61.9% on the CPU and 50.8% on the GPU, while achieving slight accuracy improvements rather than degradation.

## 1. Introduction

Video is continuous data in time; the information obtained from the current frame is less significant when conditioned on previously observed frames than when not. Bio-vision efficiently recognizes real-world video in directed time by focusing only on areas of change, “events”, and reducing redundancies inherent in a video [1]. Inspired by this, efficient sensor devices have been developed that generate signals asynchronously only when luminance changes [2]. In video compression, the most popular approach involves encoding frames in a video with luminance changes and motions from a keyframe [3].

What about online video understanding, where a computer sequentially observes frames in a directed video stream and outputs understandable information? Many methods are based on frame-level visual feature extraction at an equivalent computational cost [4,5,6]. In online video understanding, which often requires real-time processing, computational efficiency, especially in terms of inference speed, is paramount, and the largest bottleneck involves frame-level visual feature extraction. Considering the redundancy in a video, restricting the process to only the informative areas of the video conditioned on the past has the potential to significantly reduce computational costs.

Although there are many efficient online video understanding methods that account for video redundancy for specific tasks (e.g., action recognition [4,7], pose estimation [5,8]), there are only a limited number of task-agnostic methods. Skipping unchanged parts of the frame image is a simple and reasonable idea, and methods for convolutional neural networks (CNNs) have realized reductions in theoretical computation costs [9,10]. However, the overhead of unstructured memory access due to the naive implementation of convolution on sparse input has made it difficult to improve actual inference speed, even if the theoretical computational costs were reduced. Recently, a sophisticated CUDA implementation of this skipping idea has improved actual inference speed [11,12].

Meanwhile, Vision Transformer (ViT) [13], which takes an image as a sequence of patches (tokens) and analyzes them with a Transformer [14], derived from natural language processing, has emerged in recent years as the backbone for broad vision tasks [15,16,17]. ViT can strongly scale with the amount of training data due to its representational capacity, and pre-trained ViTs have demonstrated performance that rivals or surpasses CNNs as the backbone for various vision tasks, making them promising for online video understanding. The Transformer block, the basic element of ViT, operates as a set-to-set transformation. Reducing the number of tokens reduces computation without increasing memory access overhead. Focusing on these properties, we implement visual sparse processing on ViT to improve actual inference speed for online video.

**Figure 1:**
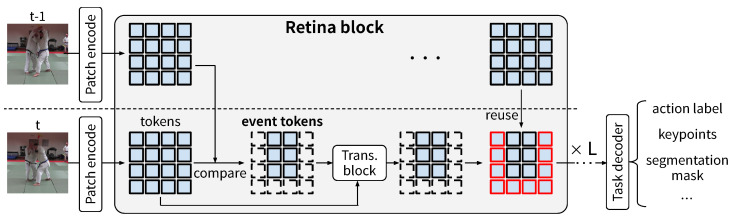
Overview of RetinaViT. Based on Vision Transformer (ViT) [13], RetinaViT converts an input frame image into tokens and processes them with a stack of Transformer blocks. The key difference is that RetinaViT detects tokens that have changed compared to those at the previous timestep in the same stage (block), referred to as event tokens. It then inputs only these event tokens as queries to the Transformer blocks for feature extraction, while reusing the previous tokens for the rest (represented by rectangles with red edges). For simplicity, this figure does not show the restriction of keys and values to the neighborhood of event tokens (see Figure 2 and Section 3.1 for details). This simple framework is task-agnostic, and RetinaViT can be used as the backbone for a wide range of online video recognition tasks.

In this study, we propose RetinaViT, which can efficiently extract visual features from online video streams by reducing the redundancy of the target video. We show an overview of RetinaViT in Figure 1. The basic concept of RetinaViT is intuitive: only the changed parts in a frame (event tokens) are processed, and for the other parts, previously processed information is reused. This simple design does not assume any specific task, making RetinaViT a task-agnostic backbone capable of extracting visual features at a wide range of granularities, from low to high levels. Specifically, we approximate the process of the Transformer block by inputting only event tokens as queries and reusing the previous information for the rest of the tokens. In addition, inspired by recently developed local attention methods [18], we further improve efficiency by restricting keys and values to the neighborhood of event tokens. Due to the nature of the transformer block, reducing the number of tokens directly leads to improvements in actual inference speed without requiring a cumbersome implementation. RetinaViT has multiple approximation parameters, and we randomly vary them during model training to create a single model that adapts to any approximation parameter setting. After training, black-box optimization is used to find parameters that make the model both accurate and computationally efficient.

Our main contribution is the proposal of RetinaViT, and we summarize its features as follows: RetinaViT is (1) a task-agnostic visual feature backbone for online video understanding that (2) leverages the strong representational power of ViTs, and (3) does not require cumbersome implementations such as custom CUDA kernels. RetinaViT is the first method to realize these features simultaneously. In our experiments, we conducted an extensive ablation study and comparison with existing methods on three online video understanding tasks, each with different characteristics, and demonstrated the effectiveness of our method in improving the trade-off between accuracy and efficiency.

## 2. Related Works

**Online video understanding.** Online video understanding is a general term for tasks that involve the observation of frames moment by moment and output understandable information (e.g., action labels in online action recognition and keypoint coordinates in online pose estimation). Processing each frame independently (i.e., using a sliding-window approach) is the most basic method and has proven to be a competitive solution for online video understanding [5,19]. The dominant approach consists of conducting visual feature extraction at each frame using a visual encoder (e.g., CNN, ViT) and a task-specific decoder, often including temporal reasoning for current and past features. For example, in action recognition, ECO [4] and TRN [7] independently extract frame-level features using 2D CNNs and then use 3D CNNs and LSTMs [20] to associate them, respectively. The recently proposed method using Transformers [14] as decoders also requires frame-level visual feature extraction before the decoder [21,22,23,24]. In online pose estimation, methods exist to extract frame-level features and relate them using LSTMs [8], warping and graph inference [25], and kernel distillation [26]. The dominant paradigm in online object tracking, tracking-by-detection [20,27,28,29,30,31], assumes frame-by-frame object detection. Therefore, frame-level visual feature extraction is fundamental in online video understanding but also accounts for a large portion of the overall computational cost. Our RetinaViT is an efficient frame-level feature extractor that has the potential to fundamentally improve the efficiency of many online video understanding tasks in a plug-and-play manner.

**Efficient neural visual backbone.** As neural visual models have been fundamental for a broad range of computer vision tasks, the development of efficient neural visual models has attracted considerable interest from researchers. One perspective categorizes approaches for improving the efficiency of neural visual models into static or dynamic. While the static approach imposes a constant computational cost regardless of the input, the dynamic approach adapts the computational cost based on the complexity of the input. Optimizing the model structure and neural operations is a common static approach. This includes techniques such as convolution decomposition in CNNs [32,33,34], local attention [18], and the decomposition of attention [35,36] in ViTs, as well as neural architecture search [37,38]. In addition, pruning [39,40,41] and quantization [42,43] also fall under the static approach. In contrast, the dynamic approach includes methods that dynamically change the computational graph, such as early termination [44,45], which changes the depth of layers to be processed based on input data. Methods that select blocks to be processed [46], dynamic quantization [47], and patch selection [48,49,50,51] also fall under this category. Dynamic approaches tend to be more complex than static approaches, as they require a policy model to make processing decisions separate from the main model [46] or additional loss functions [47]. In principle, however, dynamic approaches have a higher potential for efficiency than static approaches because they can optimize the allocation of processing costs based on the complexity and importance of the input data. Our method falls under the dynamic approach and is unique compared to these methods in that it focuses on redundancies inherent in online videos.

There are methods similar to ours that focus on video redundancy to improve efficiency. In static approaches used for online video pose estimation and online video object segmentation, methods that warp information processed in the past based on motion information reduce computational costs [8,52]. Several dynamic methods adjust computational costs (e.g., model size [53,54], quantization accuracy [55], and input resolution [54]) using a policy model based on past information. In particular, AR-Net [54], which dynamically changes the input resolution, is similar to our proposed method in terms of adaptive input adjustment; however, our method selects image patches, which allows for more granular adjustments. In addition, while all these methods assume specific tasks, our RetinaViT is a task-agnostic, efficient visual feature backbone for online video understanding.

The study of processing convolution only at the point of change is most relevant to our method. Implementing sparse convolution with actual inference speed improvement is not easy due to memory access overhead [9,10,56,57,58], while DeltaCNN [11] achieves actual inference speedups on GPUs through an elaborate CUDA implementation. MotionDeltaCNN [12] further improves the efficiency of DeltaCNN by using correspondence points under conditions of known camera motion. Unlike these methods, our approach is built on ViT, which has recently demonstrated effectiveness across a variety of computer vision tasks. Our method provides actual speedup on both CPUs and GPUs without requiring complex implementation, thanks to the nature of ViT, where the simple reduction of tokens directly reduces the actual computational cost.

Although there are recent studies that conduct token reduction in ViT to improve the efficiency of video processing, most of them [59,60,61,62] are designed for offline video processing, where all frames, including future ones, are accessible. This is different from online video processing, which we focus on. A concurrent study [63] conducts token reduction for online video recognition, but it is limited to the object tracking task, as tokens are selected based on past detection results. In contrast, our method is task-agnostic and can be used as the backbone for a wide range of online video understanding tasks due to its simplicity.

## 3. Method

We propose RetinaViT as an efficient frame-level visual feature extractor for online video. RetinaViT is composed of Retina blocks that approximate the original Transformer block to process only tokens that have changed over time, called event tokens. RetinaViT has several approximation parameters that adjust computational costs. During training, we randomly vary the approximation parameters and obtain a single model that can extract the appropriate features with any setting of approximation parameters. This training procedure is also expected to have a regularization effect, as in previous work for image recognition [64]. The approximation parameters of the trained RetinaViT are then tuned using black-box optimization to maximize accuracy and computational efficiency.

In the following, we will first describe the approximation of the Transformer block (Section 3.1), and then the model training and tuning of the approximation parameters (Section 3.3).

### 3.1. Approximation of Transformer Block

**Transformer block basics.** ViT [13] divides an image into non-overlapping patches, encodes them as token embeddings via linear projection and positional embedding (i.e., patch encoding; see Appendix A), and analyzes them with Transformer blocks [14]. Let us denote token embeddings as $z_{l}\in R^{N\times D}$, where *N* is the number of tokens, *D* is the number of channels, and l∈{0,1,…,L} is the depth in ViT, which consists of *L* Transformer blocks. Here, ($z_{0}$ is the output from the initial patch encoding, while $z_{l>0}$ is the output of the *l*-th Transformer block). In the *l*-th Transformer block, the token sequence is processed as follows:

(1) $$\begin{array}{c} q_{l+1}=k_{l+1}=v_{l+1}=\mathrm{LN}\left(z_{l}\right), \end{array}$$

(2) $$\begin{array}{c} h_{l+1}=\mathrm{MHA}\left(q_{l+1},k_{l+1},v_{l+1}\right), \end{array}$$

(3) $$\begin{array}{c} z_{l+1}=\mathrm{MLP}\left(\mathrm{LN}\left(h_{l+1}\right)\right)+h_{l+1}, \end{array}$$

where LN is layer normalization [65] (Appendix B), MHA is multi-head attention [14] (Appendix C), and MLP is a two-layer token-level MLP, including the GELU activation function [66] with a hidden size of 4D. q, k, and v correspond to the queries, keys, and values that are inputs to multi-head attention, respectively (Appendix C). The original Transformer block uses what is known as self-multi-head attention, where queries, keys, and values are all identical, as shown in Equation (1) [13]. We refer to keys and values as “contexts”.

**Retina block.** In videos, pixel values are often very similar within the spatiotemporal neighborhood. The same tendency holds for the feature space, where features for the same spatial location are often similar. We introduce a method for the efficient approximation of the Transformer block by processing only tokens with significant changes from the previous frame—event tokens—and skip the processing of the remaining tokens, which we call non-event tokens.

**Figure 2:**
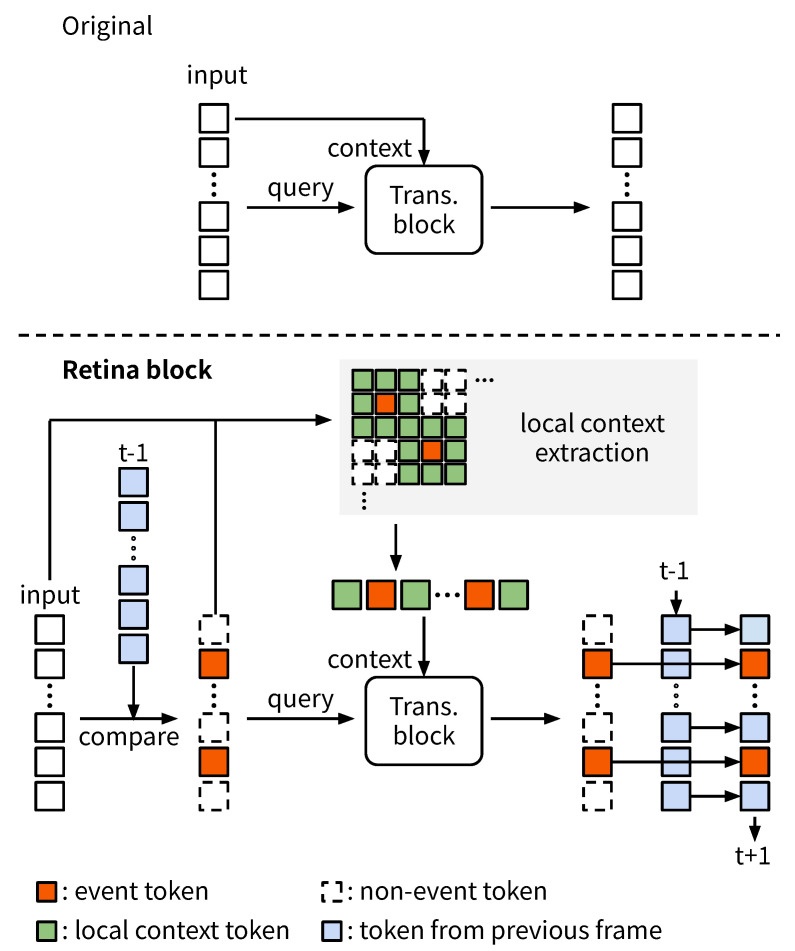
Original Transformer block (**top**) and Retina block (**bottom**). Each rectangle represents a token. In the Retina block, only event tokens, i.e., tokens that have changed over time, are input as queries, and the previous information is reused for the rest. In addition, by restricting the context tokens to the spatial neighborhood of the event tokens, the computational cost is further reduced.

We refer to this approximated Transformer block as “RetinaBlock” and provide an overview of its process in Figure 2. First, we introduce the “event score”, which is the L2 distance between a token in the current frame and its spatial counterpart in the previous frame, and determine event tokens by thresholding this score. The indices of event tokens in the *l*-th token sequence $z_{l}^{t}$ at time *t* are determined as follows:

(4) $$\begin{array}{c} S_{l}^{t}=\left\{i\in \left\{1,\ldots ,N\right\}|i\leq N_{s}\vee d_{t,(i)}^{l}<\delta_{l}\right\}, \end{array}$$

(5) $$\begin{array}{c} d_{l,(i)}^{t}=\left|\right|z_{l,(i)}^{t}-z_{l,(i)}^{t-1}{\left|\right|}_{2}, \end{array}$$

where $z_{l,(i)}^{t}$ is the *i*-th token in the token sequence $z_{l}^{t}$, $d_{l,(i)}^{t}$ is the event score, and $N_{s}$ is the number of special tokens (e.g., classification token [13]) that are not derived from the input image patch. These tokens are always treated as event tokens.

In the Retina block, Equations (1)–(3) are modified as follows:

(6) $$\begin{array}{c} q_{l+1}=\mathrm{LN}\left(z_{l,i\in S_{l}^{t}}^{t}\right),\;k_{l+1}=v_{l+1}=\mathrm{LN}\left(z_{l}^{t}\right), \end{array}$$

(7) $$\begin{array}{c} h_{l+1}=\mathrm{MHA}\left(q_{l+1},k_{l+1},v_{l+1}\right), \end{array}$$

(8) $$\begin{array}{c} z_{l+1,i\in S_{l}^{t}}^{t}=\mathrm{MLP}\left(\mathrm{LN}\left(h_{l+1}\right)\right)+h_{l+1}, \end{array}$$

(9) $$\begin{array}{c} z_{l+1}^{t}=\mathrm{Align}\left[z_{l+1,i\in S_{l}^{t}}^{t},z_{l+1,i\in \bar{S_{l}^{t}}}^{t-1}\right], \end{array}$$

where Align[·] denotes the concatenation of token sequences and their alignment to the original token order. Multi-head attention only takes event tokens as queries and all tokens as contexts (Equation (7)), followed by a token-level MLP (Equation (8)).

In multi-head attention, each query token is processed independently of other query tokens, relying solely on itself and the contexts. Similarly, the MLP processes each token independently. Therefore, the outputs for event tokens are identical to those processed by the vanilla Transformer block. By combining the event tokens with the non-event tokens, which are carried over from the previous timestep, we obtain the final output (Equation (9)).

Since the theoretical computational complexity of the Transformer block is (roughly) proportional to the number of query tokens [64,67], the computational cost can be reduced by increasing the number of non-event tokens.

We construct RetinaViT by stacking multiple Retina blocks and train it end-to-end. Once a token becomes a non-event token in RetinaViT, it never reverts to an event token because its difference with the corresponding token at the previous time is zero (as it is replaced by itself). On the other hand, even if a token is an event token in an earlier block, as the feature extraction progresses, the event score decreases, and the token may become a non-event token. This can be seen as an adaptive adjustment of the number of blocks to process for each token.

Note that at the first timestep t=0, any tokens are not dropped, i.e., all tokens are processed, and their information is reused in subsequent timesteps.

**Local context tokens.** In frame images, spatially close areas are more strongly related than distant areas. Following this characteristic, there are methods to improve the efficiency of Vision Transformers (ViTs) by limiting attention to spatially local areas [18,68]. Similarly, we consider restricting attention to the spatial locality around each event token to achieve a more aggressive reduction in computational costs. However, existing local attention methods are designed for densely arranged tokens and cannot effectively improve inference speed for sparse tokens due to memory access costs. Implementing efficient local attention for sparsely arranged event tokens is not trivial.

We introduce local attention to the Retina block with a simple yet practically effective implementation. Specifically, we consider the union set of spatial neighborhoods of event tokens as the context tokens (see Figure 2). Equation (1) is, thus, modified as follows:

(10) $$\begin{array}{c} k_{l+1}^{t}=v_{l+1}^{t}=\mathrm{LN}\left(z_{l,\phi_{m_{l}}\left(S_{t}^{l}\right)}^{t}\right), \end{array}$$

where $\phi_{m_{l}}$ is a function that extends the index to a spatial neighborhood, and $m_{l}$ is the number of neighbors in the *l*-th block.

In existing local attention methods [18,68], only the neighboring tokens of each token are used as context tokens. In contrast, our method includes the neighboring tokens of other event tokens as well, thus broadening the context for each event token. This approach leaves room for theoretical reductions in computational costs. Although memory-access overhead is minimized and the number of contexts correlates with theoretical computational costs, this implementation is effective enough to practically improve computational efficiency.

### 3.2. RetinaViT

RetinaViT is a stack of multiple Retina blocks as shown in Figure 1. We construct multiple variants of RetinaViT by varying the size of the Transformer block in the Retina block while keeping the patch size at 16×16, as summarized in Table 1. Following the original naming convention, we refer to RetinaViT variants using the name of the architecture and the patch size; for example, RetinaViT-B16 refers to the “Base” model with a patch size of 16×16. Each variant of RetinaViT has the same Transformer block configuration as the corresponding original ViT (e.g., RetinaViT-B16 corresponds to ViT-B16); therefore, it is possible to transfer the weights of the corresponding pre-trained ViT to RetinaViT.

For downstream tasks, we can extract frame-level features in multiple ways (all tokens or classification tokens) at multiple stages (final layer or intermediate layers) from RetinaViT. In the experiments, we selected the appropriate method for each downstream task, following the standard practice of the task and the decoder used. Details are described in Section 4.1.

### 3.3. Model Training and Approximation Parameter Optimization

RetinaViT has approximation parameters to adjust computational costs, including thresholds ${\left\{\delta_{l}\right\}}_{l=1}^{L}$ for the event score and the neighborhood numbers of context tokens ${\left\{m_{l}\right\}}_{l=1}^{L}$. We determine these approximation parameters by a train-then-optimize strategy; we first train RetinaViT so that it can extract appropriate features with any approximation parameter setting, and then fix the trained model and optimize the approximation parameters using black-box optimization to maximize both accuracy and efficiency. The details of training and optimization are as follows.

**Model training.** We randomly vary the approximation parameters $\theta =\{{\left\{\delta_{l}\right\}}_{l=1}^{L},{\left\{m_{l}\right\}}_{l=1}^{L}\}$ over a defined range during training, obtaining a model that can extract features appropriately with any approximation parameter setting in that range. $m_{l}$ varied as an integer in the range [0,4]. $\delta_{l}$ depends on the distribution of the event score, which varies during training. Therefore, we pick up tokens with event scores in the *r* percentile as event tokens. We set *r* in the range [0,1].

In this way, the model undergoes various approximation parameter settings during training and can properly infer under any of these settings. Our training method is the same as standard training except for the random variation of these parameters and does not require any additional losses other than the main loss. As in previous work for image recognition [64], this training procedure is also expected to have a regularization effect.

**Approximation parameters optimization.** After model training, we obtain the approximation parameters θ of the trained model $F_{\theta }\left(\cdot \right)$ by maximizing accuracy and computational efficiency using black-box optimization. We solve the following multiobjective optimization:

(11) $$\begin{array}{c} \underset{\theta }{\mathrm{maximize}}\;\left(P,\;-C\right), \end{array}$$

$$\begin{array}{c} P=P\left(F_{\theta }\left(\cdot \right),D_{\mathrm{train}}\right),\;C=E_{\left(x,y\right)∼D_{\mathrm{train}}}\;\left[C(F_{\theta }\left(\cdot \right),x)\right], \end{array}$$

where $D_{\mathrm{train}}$ is the training data, *x* is the input and *y* is the target in the training data, respectively. $P(F_{\theta }\left(\cdot \right),D_{\mathrm{train}})$ is a task-specific evaluation metric when applying $F_{\theta }\left(\cdot \right)$ to $D_{\mathrm{train}}$ (e.g., frame-level accuracy for online action recognition, IoU for video object segmentation), and $C(F_{\theta }\left(\cdot \right),x)$ is the computational cost (e.g., FLOPs, runtime) when *x* is processed by $F_{\theta }\left(\cdot \right)$. In real application cases, the optimization problem can be modified according to the scenario’s requirements; for example, one can formulate a constrained optimization that maximizes accuracy by setting an upper bound on computational cost. Notably, in our approach, once model training—the most time-consuming process—is complete, we can obtain models with different computational costs by changing the constraints and optimizing the approximation parameters. In the experiments, we solve the above multiobjective optimization using MOTPE [69] and draw the Pareto front between accuracy and efficiency.

## 4. Experiments

We conducted extensive evaluations on three online video understanding tasks to assess the effectiveness of RetinaViT. The rest of this section is organized as follows: first, we describe the setup for the three evaluation tasks in Section 4.1, followed by the task-agnostic implementation details in Section 4.2. Next, we provide the evaluation results for each component of RetinaViT, the analysis of the training procedure, and a comparative study with existing methods in Section 4.3, Section 4.4 and Section 4.5, respectively. Finally, we provide visualizations of event scores, event tokens, and predictions to discuss the behavior of RetinaViT in Section 4.6.

### 4.1. Task Setups

RetinaViT can be applied as a frame-level visual feature encoder for a variety of online video understanding tasks. To demonstrate its effectiveness and generalizability, we evaluate RetinaViT on the following three tasks, each with different characteristics.

**Online action recognition** is the task of classifying the classes of actions taking place at each frame in an online video. We used the 50Salads dataset [70], which contains 50 videos in which 25 people prepare two kinds of mixed salads. The videos, each consisting of 9000 to 18,000 RGB frames, are annotated with 17 action classes per frame. We built RetinaViT-T16/S16/B16 based on the original ViTs pre-trained on ImageNet-21k [71,72]. Each frame was processed independently in a sliding-window manner, and we used the classification token as the frame-level feature. The final classification result for each frame was obtained by inputting the frame-level feature into a linear decoder. Although we could use more sophisticated decoders (e.g., LSTM, Transformer) that take temporal information into account, we used the simplest one to measure the pure performance of feature encoders. We used cross-entropy as the loss function. Following previous works [73], we downsampled the videos in the 50Salads dataset from 30 fps to 15 fps. The video was divided into non-overlapping chunks of 20 frames. For training, the frames were cropped randomly but consistently throughout each chunk and resized to 224×224. For testing, the frames were center-cropped and resized to 224×224. We set the maximum number of epochs to 25 and the batch size to 8. We used Top-1 accuracy as the evaluation metric.

**Online pose estimation** is the task of predicting the key point coordinates of a person in each frame of an online video. We evaluated Sub-JHMDB [74], which is a collection of 11,200 frames from 316 video clips, labeled with 15 body joints.

The video was divided into non-overlapping chunks of 20 frames. As in previous studies [8,10,25,26], we cropped the frame images based on the bounding box of the person obtained from the puppet masks in the dataset. The cropped images were then resized to 256×192 for model input. A random flip was performed for data augmentation during training. Models were required to output the keypoint coordinates of the person from these cropped frame images. We set the maximum number of epochs to 200 and the batch size to 4. We adopted the PCK introduced in [75] as a metric. Each predicted coordinate was considered correct if it was within α·max(h,w) from the true position, where *h* and *w* are the height and width of the bounding box. We set α to 0.2.

We built RetinaViT-S16/B16 based on ViTPose [15] MS COCO [76]. In ViTPose, the original ViTs were used as the backbone; therefore, we could transfer the weights of the ViT part to RetinaViT. Following the original ViTPose, we used the output of the last block as the frame-level feature and upsampled it with the decoder consisting of three deconvolution layers to output keypoint coordinates in heatmap format. We used the mean squared error on heatmaps as the loss function.

**Video object segmentation** is a task in which the model outputs the segmentation mask of the object in each frame, given the segmentation mask for the first frame in an online video. For evaluation, we used DAVIS 2017 [77], which contains 60 training videos, 30 validation videos, and 30 test-dev videos. We constructed the methods based on BMVOS [6], which consists of multi-scale frame-level feature extraction using DenseNet and a decoder that considers the relationship between frame features. We conducted the evaluation by replacing the DenseNet encoder with RetinaViT-T16/S16, which were based on the pre-trained ViTs on ImageNet-21k as in the action recognition task. For multi-scale frame-level feature extraction, we upsampled the output of the 4th, 8th, and 12th blocks to different resolutions to fit the BMVOS decoder with deconvolutions, as conducted in DPT [16]. We set the maximum number of epochs to 100 and the batch size to 8. We cropped the region randomly but consistently throughout each video sequence and resized it to 384×384 during training, and resized the frame to a height of 384 while maintaining the aspect ratio during testing.

Following the previous work [6], we used the G score, which is the average of the segment intersection-over-union (IoU) and the boundary IoU, for evaluation [77].

### 4.2. Implementation Details

We used Adam [78] for training on all three tasks with a learning rate of $10^{-4}$. To tune the approximation parameters, we used the metric function of each task as the performance metric J(·) and GPU inference time as the computational cost B(·). Although FLOPs can be used as a measure of computational cost, optimizing for actual inference time produced a better trade-off, as the actual inference time has some overhead that cannot be measured by FLOPs. In real-world applications, it should be possible to measure and optimize inference time for each device actually used to achieve a better trade-off. While our method also supports constrained optimization of computational cost, which is thought to be useful in real applications, we solved the multiobjective optimization in Equation (11) to obtain the Pareto front using MOTPE [69] for a detailed evaluation of the trade-off between accuracy and efficiency. Specifically, we first obtained the Pareto front on the training split by optimization with 30 trials, then drew the Pareto front on the test (or validation) split by evaluating the test (or validation) split using the approximation parameters of each point of the Pareto front on the training split. Note that in this phase, the model was in inference mode, so one trial was faster than one epoch in model training. Unless otherwise specified, we applied event token detection at the first and fourth blocks, as determined by the ablation study (Section 4.3).

The actual inference time was measured on both a CPU and a single GPU. We used an Intel(R) Core(TM) i9-10980XE CPU @ 3.00 GHz (Intel Corporation, Santa Clara, CA, USA) for the CPU and an NVIDIA RTX A5000 (NVIDIA Corporation, Santa Clara, CA, USA) for the GPU. For inference on the CPU, we set the batch size to 1. For the GPU, a small batch size did not make a significant difference in inference time because the processing power of the device could not be fully utilized (the same was reported in [11]), so we set the batch size to 64 and reported the average per sample inference time.

### 4.3. Ablation Study

**Sanity check.** To confirm the basic validity of the proposed method, we compare it with systematically constructed baselines on the action recognition task. Specifically, we consider multiple patterns in the training and inference phases. First, for training, in addition to our (1) event-based approach, where tokens are dropped based on event score and randomly sampled thresholds, the following can be considered: (2) vanilla: the training strategy used in the original ViT (i.e., tokens are not dropped), and (3) random: a training method that ignores event score and drops tokens completely at random. For inference, we consider our (1) event-based approach, where event tokens are selected by thresholding the event score, and (2) random, where event tokens are selected completely at random.

**Figure 3:**
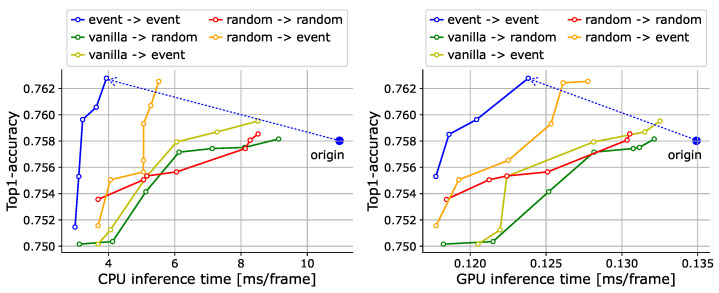
Sanity check on 50Salads [70]. We present trade-off curves between the accuracy and inference time on the CPU (**left**) and GPU (**right**). In the legends, the start and end points of each arrow represent training and inference strategies, respectively. “Origin” in each graph represents the result where no token selection is used (original ViT). We draw dashed arrows representing the improvements in the trade-off between our method (event-based -> event-based) and the corresponding “origin”.

The results are shown in Figure 3. First, we can see that our method achieves a significant speed increase over the original ViT with accuracy improvements (rather than degradations). We speculate that this is due to the improved robustness of focusing only on the changed areas and the regularization effect of randomly dropping tokens, as in [64]. Second, when training with the “vanilla” approach, we observe an accuracy drop as the number of tokens decreases because the model is not trained to handle token drops. On the other hand, the model trained with the “random” strategy assumes token drops, leading to some improvement in the trade-off during “event-based” inference, but it is not as effective as the proposed method. These comparisons indicate that the event score is meaningful for determining the importance of a token.

**Figure 4:**
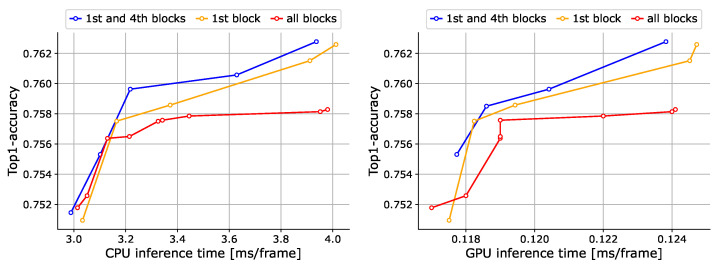
Ablation results for the locations of event token detection on 50Salads [70]. We show trade-off curves between the accuracy and inference time on the CPU (**left**) and GPU (**right**). Note that we do not show “origin” (original ViT) in this figure to clarify the differences, but we have successfully improved the trade-off significantly compared to the “origin” as shown in Figure 3.

**Locations of event token detection.** The detection of event tokens can be conducted in any block of RetinaViT. However, if event token detection is performed in too many blocks, the number of event tokens may become extremely small during training, which could be problematic. Therefore, we compared the cases in which event tokens are detected in only the first block, the first and fourth blocks, and all blocks. As can be seen from Figure 4, the accuracy deteriorates when event token detection is performed in all blocks. We believe this is due to the extremely small number of tokens that the model can consider (i.e., event tokens) during training, as noted above. Although the case involving the first block alone recorded a decent trade-off, the case involving the first and fourth blocks, in which event tokens could be determined at multiple phases, was found to have the best trade-off.

**Figure 5:**
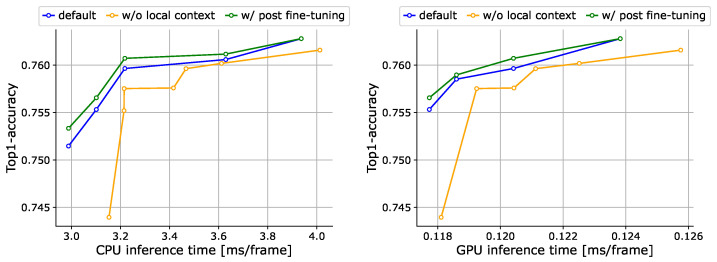
Ablation results for local context tokens and post-fine-tuning on 50Salads [70]. We show trade-off curves between the accuracy and inference time on the CPU (**left**) and GPU (**right**). Note that we do not show “origin” (original ViT) in this figure to clarify the differences, but we have successfully improved the trade-off significantly compared to the “origin” as shown in Figure 3.

**Local context tokens.** We compare RetinaViT with and without local context tokens in Figure 5. We can see that the trade-off is improved by introducing local context tokens. The introduction of locality in ViT, as per existing studies [18,68], is found to be effective in terms of increasing efficiency.

**Fine-tuning after approximation parameters optimization.** We have the option of further fine-tuning after optimizing approximation parameters, as is conducted in pruning methods [40,79], namely post-fine-tuning. The evaluation results of this option are shown in Figure 5. While post-fine-tuning improves accuracy, the gain is not large. The model trained by our method has already undergone all possible approximation parameter settings and is, therefore, ready to work without post-fine-tuning. Since post-fine-tuning for each approximation parameter setting is labor- and computation-intensive, we did not use it in the following experiments.

### 4.4. Analysis of the Training Procedure

**Figure 6:**
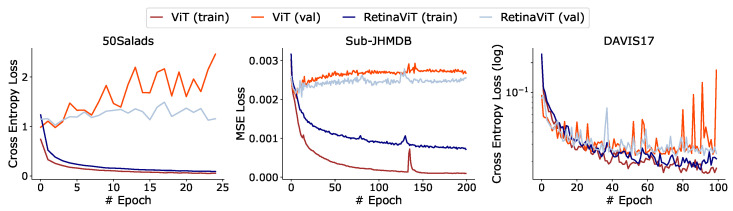
Comparisons of the learning curves between RetinaViT-S16 and the original ViT-S16. The vertical axis represents the loss for each task, and the horizontal axis represents the number of epochs. In all datasets, the validation learning curves of RetinaViT are relatively stable.

The training of RetinaViT can be considered as a form of random information dropping, which may have a regularization effect, as reported in previous studies [64,80,81]. To investigate this, we compared the learning curves of RetinaViT with those of the corresponding original ViT in Figure 6. We can see that the learning curves on the validation sets of RetinaViT are relatively stable across all datasets. This regularization effect is considered to be one of the reasons for the slight performance improvement over the original ViTs (Figure 3).

### 4.5. Comparative Study with Existing Methods

**Figure 7:**
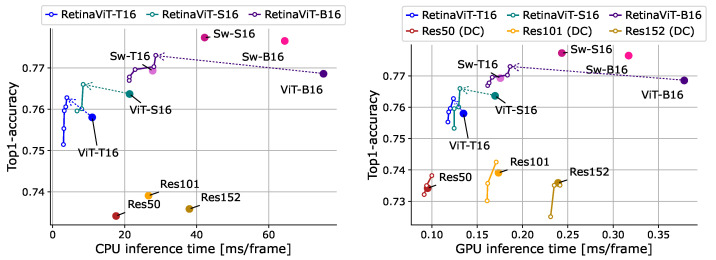
Comparisons of the trade-off between the accuracy and inference time on 50Salads [70] (val). Inference time was measured on the CPU (**left**) and GPU (**right**). “Sw” and “Res” represent Swin Transformer [18] and ResNet [82], respectively. “DC” represents DeltaCNN [11], which we only use on the GPU as it does not support CPU inference. Dashed arrows represent the improvements in the trade-off between RetinaViT and the corresponding original ViT.

**Online action recognition.** We implemented the widely used visual backbones ResNet-50/101/152 [82], Swin Transformer-T16/S16/B16 [18], ViT-T16/S16/B16 [13], and RetinaViT-T16/S16/B16 for comparison, and ResNets are pre-trained on ImageNet and the others are pre-trained on ImageNet-21k. All models are used as frame-level classifiers without any special decoder, such as LSTM. We also applied DeltaCNN [11], a method for CNNs that processes only those parts of the frame that have changed, similar to the proposed method, to ResNets. Since there is only a CUDA implementation of DeltaCNN, we evaluated it only on a GPU. DeltaCNN, like our method, has thresholds that adjust the computational costs, and we determined them using the same black-box optimization procedure as RetinaViT to ensure a fair comparison. The comparisons are shown in Figure 7. First, compared to original ViTs, RetinaViT achieved significant improvements in efficiency with better accuracy and the improvement in efficiency is more pronounced when the model is larger. In particular, when we used ViT-B16 as a base model, RetinaViT achieved 61.9% and 50.8% reductions in inference time with slight accuracy improvements rather than degradation in action recognition. For ViT-T16, which is the smallest model, the efficiency improvement on the GPU is smaller. We attribute this to the fact that the GPU’s computational capacity is sufficiently large compared to the model’s computational cost, making the difference insignificant. As noted in Section 4.3, we attribute the improvement in accuracy to the improved robustness by focusing on changed locations and the regularization effect of random token dropping. Due to the strong representational capabilities of the base models, ViTs, RetinaViT offers a better trade-off compared to ResNets and ResNets equipped with DeltaCNN. While Swin Transformers show strong trade-offs, RetinaViT achieves comparative accuracy even in the low-computational-cost range (0.25 ms/frame in GPU inference time, 20 ms/frame in CPU inference time), where the smallest model of Swin Transformers, T16, cannot be reached. We believe that RetinaViT can be the best option for applications that require such a low computational cost range.

**Figure 8:**
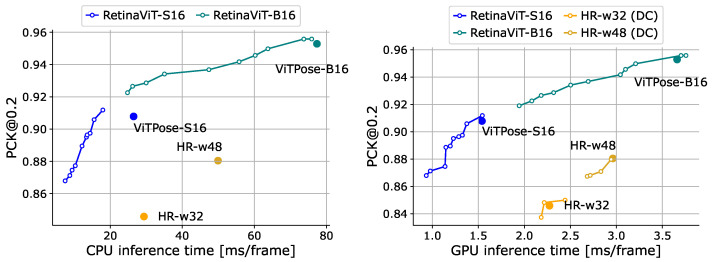
Comparisons of the trade-off between accuracy (PCK@0.2 [75]) and inference time on Sub-JHMDB [74] (val). Inference time was measured on the CPU (**left**) and GPU (**right**). “HR” represents HR-Net [83]. “DC” represents DeltaCNN [11], which we only use on the GPU as it does not support CPU inference.

**Online pose estimation.** We compare our RetinaViT with ViTPose-S16/B16 (which are the base models of RetinaViT) and HR-Nets (w32, w48) [83]. We also introduce DeltaCNN to HR-Nets, as we did with the action recognition evaluation. All models were pre-trained on MS COCO [76]. We show the results in Figure 8. RetinaViT achieved higher efficiency with equal or better accuracy than their respective base models (ViTPose), especially on the CPU, and demonstrated a superior trade-off compared to HR-Nets and HR-Nets equipped with DeltaCNN. Compared to the case of action recognition, the improvements from the base models were not large. This is likely because dropping tokens more strongly affects accuracy due to the nature of pose estimation, where fine-grained features are more crucial to predict keypoint coordinates, which we also discuss in Section 4.6.

**Figure 9:**
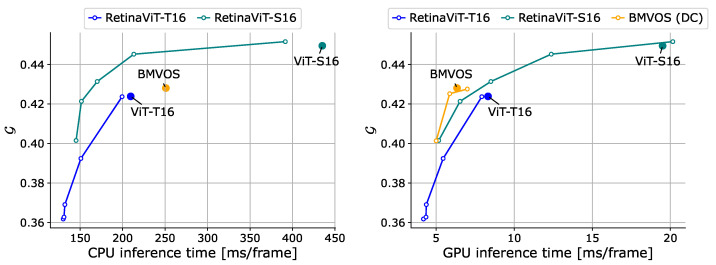
Trade-off comparisons between the accuracy (G score [77]) and inference time on DAVIS17 [77] (test-dev). Inference time was measured on the CPU (**left**) and GPU (**right**). “DC” represents DeltaCNN [11], which we only use on the GPU as it does not support CPU inference.

**Video object segmentation.** Finally, we evaluated VOS, replacing the frame encoder of BMVOS. The detailed settings are described in Section 4.1. We show the results in Figure 9. We can see that the model significantly improves computational efficiency without severe accuracy degradation, especially when using ViT-S16. Even though VOS requires more fine-grained features than pose estimation, RetinaViT shows a certain effectiveness. The reason why the computational efficiency does not improve much with ViT-B16 is that the model’s computational cost is too small compared to the device’s computational capacity, making the impact of token reduction on inference speed small, as mentioned in online action segmentation. Compared to BMVOS, RetinaViT shows a comparable trade-off. Given that ViTs scale better in performance with the training data numbers compared to CNNs, and that larger ViT-based pre-trained models are expected to emerge in future research, we would like to emphasize the importance of improving trade-offs in ViTs.

### 4.6. Visualization

**Figure 10:**
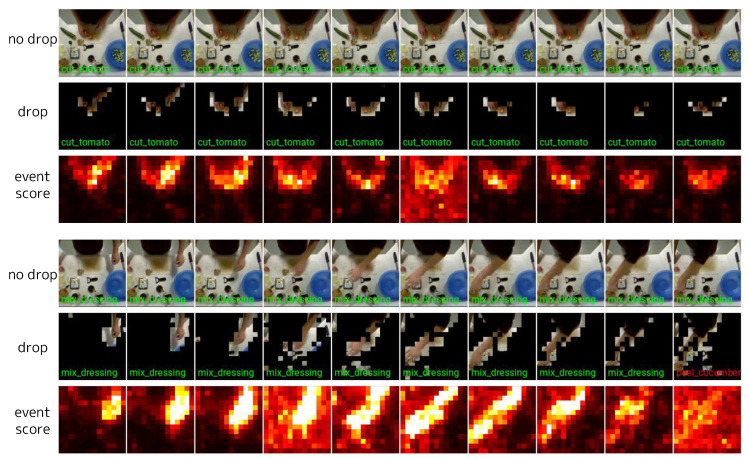
Visualization of event scores, event tokens, and predictions on 50Salads [70]. The frames are arranged from left to right in time order, and each column represents the same timestep. “no drop” represents the prediction without dropping tokens (i.e., $\delta_{l}=0$ for all *l*) overlaid on the input frames. “drop” represents the event tokens in the fourth block, on which the corresponding prediction is overlaid. For visibility, we overlaid the locations of event tokens on the corresponding input RGB frames, where the non-event tokens are blacked out. The predictions are drawn in green if correct and in red if incorrect. “event score” represents the event scores as a heatmap at the input for the fourth block. Note that all tokens are processed in the first frame of each video clip, which is not depicted in the figure.

**Figure 11:**
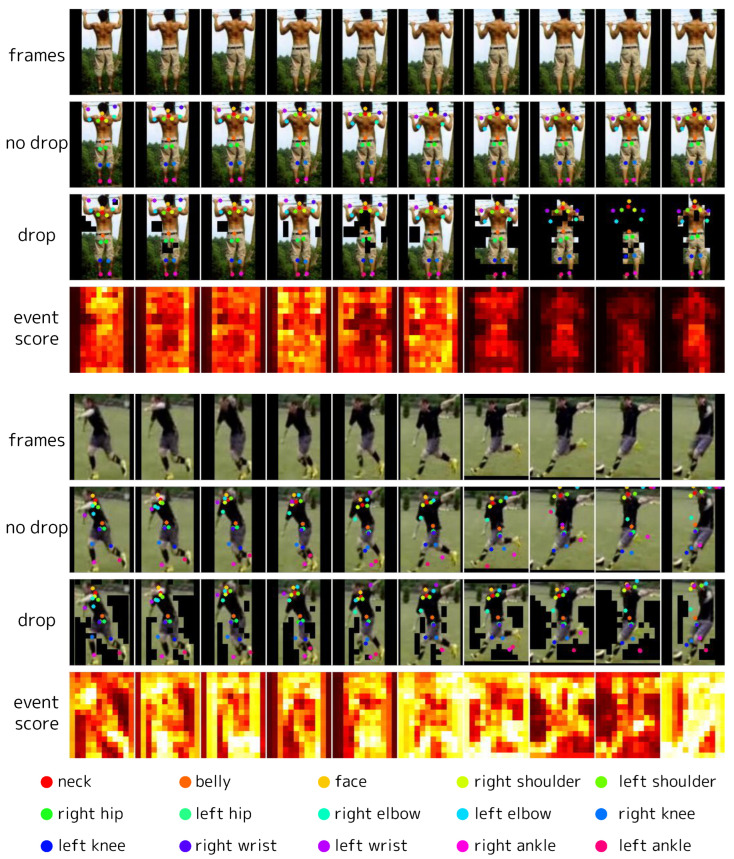
Visualization of event scores, event tokens, and predictions on Sub-JHMDB [84]. The frames are arranged from left to right in time order, and each column represents the same timestep. “no drop” represents the prediction (the locations of the key points) without dropping tokens (i.e., $\delta_{l}=0$ for all *l*), overlaid on the input frames. “drop” represents the event tokens in the fourth block, on which the corresponding prediction is overlaid. For visibility, we overlaid the locations of event tokens on the corresponding input RGB frames, where the non-event tokens are blacked out. “event score” represents the event scores as a heatmap at the input for the fourth block. Note that all tokens are processed in the first frame of each video clip, which is not depicted in the figure.

**Figure 12:**
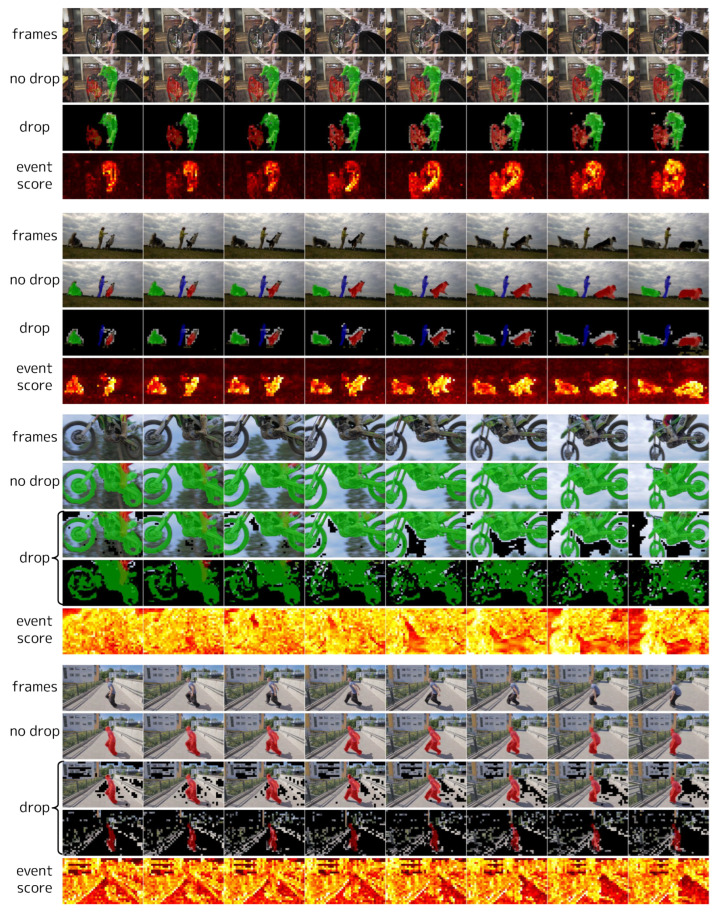
Visualization of event scores, event tokens, and predictions on DAVIS2017 [77]. The frames are arranged from left to right in time order, and each column represents the same timestep. “no drop” represents the prediction masks without dropping tokens (i.e., $\delta_{l}=0$ for all *l*), overlaid on the input frames. “drop” represents the event tokens in the fourth block, on which the corresponding prediction is overlaid. For visibility, we overlaid the locations of event tokens on the corresponding input RGB frames, where the non-event tokens are blacked out. The prediction masks are drawn in different colors for each instance. “event score” represents the event scores as a heatmap at the input for the fourth block. For the last two videos, we show two “drops” with different thresholds $\delta_{l}$ as examples where it is difficult to reduce computational costs while maintaining accuracy due to large camera motion. Note that all tokens are processed in the first frame of each video clip, which is not depicted in the figure.

We visualize event scores, event tokens, and predictions in videos from 50Salads [70] (Figure 10), Sub-JHMDB [74] (Figure 11), and DAVIS2017 [77] (Figure 12). In 50Salads, we observe that many tokens have low event scores, and dropping them does not degrade accuracy. For Sub-JHMDB, there are fewer tokens with significantly low event scores compared to 50Salads. This is primarily due to the large motions of the subjects and the perturbation caused by frame-level cropping. Although there is no significant degradation in predictions within these samples, the more aggressive token reduction can degrade accuracy, limiting the reducible computational costs. For DAVIS2017, in the top two videos, many tokens have low event scores, allowing a large number of tokens to be reduced without degrading the predictions. However, in the bottom two videos, the event scores are generally high due to large camera motions. We visualize predictions with two different thresholds for these videos (see the “drop” in the last two videos in Figure 12). When attempting to reduce a large number of tokens (similar to the top two samples) in these videos, the target objects are also dropped, causing significant accuracy degradation (see the second “drop” in each video).

We are interested in exploring frame-to-frame correspondence to mitigate the effects of camera motions and the perturbation caused by cropping in future work. Additionally, instead of a hard decision (event token or not), a method that determines the weight of processing in a multi-step or continuous manner according to event scores has the potential to address “subtle” events caused by rapid camera motions and frame-level cropping.

## 5. Conclusions

We propose RetinaViT, which efficiently extracts frame-level visual features in online video streams by exploiting redundancy in videos. RetinaViT is task-agnostic, leverages the strong representational power of ViTs, and achieves improvements in actual inference speed. We conducted extensive experiments on action recognition, pose estimation, and object segmentation in online videos, demonstrating that RetinaViT improves the trade-off between accuracy and efficiency.

In the future, for tasks involving fine-grained features, such as pose estimation and object segmentation, we are interested in investigating methods to exploit frame-to-frame correspondence to mitigate the effects of camera motions and the perturbation caused by cropping. Additionally, methods to complement excluded information (i.e., non-event tokens), such as merging them at the end of the model and using a soft definition of event tokens, might be effective. Although this study focused on a task-agnostic frame-level feature encoder, it is valuable to modify the decoder of each task to leverage event tokens for further improvement in computational efficiency. A meaningful direction will be to extend RetinaViT to ViT-based space-time visual backbones [35,36] for clip-level features.

## Figures and Tables

**Table 1:** RetinaViT variants. The number of blocks (*L*) and the configuration of the Transformer block (the number of channels *D* and the number of heads *H*) are shown.

Model Name	# Blocks (*L*)	# Channels (*D*)	# Heads (*H*)	# Params
Tiny	12	192	3	5.7 M
Small	12	384	6	22.1 M
Base	12	768	12	86.6 M

## Data Availability

The datasets used in this study are openly available in reference number [70,71,72,74,77]. The original contributions presented in this study are included in the article and further inquiries can be directed to the corresponding author.

## Abbreviations

The following abbreviations are used in this manuscript:

CNN	convolutional neural network
ViT	Vision Transformer
FLOPs	floating point operations
GPU	graphics processing unit
CPU	central processing unit
IoU	intersection-over-Union

## Appendix A. Patch Encoding and Special Tokens in ViT

Before the Transformer blocks process, ViT [13] converts an input image into a token sequence, called patch encoding. Firstly, an input image $x\in R^{H\times W\times C}$ is divided into patches and reshaped into $x_{p}\in R^{N\times (P^{2}C)}$, where *H*, *W* are the height and width of the image, *C* denotes the channel, *P* denotes the side length of each patch, and $N=HW/P^{2}$ denotes the resulting number of patches. Subsequently, each patch is mapped to a *D* dimensional feature via a token-level linear projection $\phi :R^{C}\mapsto R^{D}$ and the corresponding positional embeddings are added to it:

(A1) $$\begin{array}{c} z_{p,(i)}=\phi \left(x_{p,(i)}\right)+e_{\mathrm{pos},(i)}, \end{array}$$

where *i* is an index of the token, $x_{p,(i)}$ is the *i*-th element of $x_{p}$, and $e_{\mathrm{pos},(i)}$ is the *i*-th element of the positional embeddings $e_{\mathrm{pos}}\in R^{N\times D}$.

Finally, we concatenate ${\left\{z_{p,(i)}\right\}}_{i=1}^{N}$ and special tokens at the token axis to obtain the input to the first Transformer block. In the original ViT, which we used as our base model, a trainable classification token $z_{\mathrm{cls}}\in R^{1\times D}$ is added as a special token, as follows:

(A2) $$\begin{array}{c} z_{0}=\mathrm{Concat}\left[z_{\mathrm{cls}},z_{p,(1)},z_{p,(2)},\ldots ,z_{p,(N)}\right]. \end{array}$$

## Appendix B. Layer Normalization

We use layer normalization (LN) [65] as in the original Transformer [14]. As stated in [81], LN in the Transformer is the same as channel normalization [85], and we follow it. Given an input tensor $x\in R^{N\times D}$ (*N* is the number of tokens and *D* is the number of channels), LN normalizes the tensor over the channel axis and scales and shifts it by trainable parameters:

(A3) $$\begin{array}{c} y_{i,j}=\frac{x_{i,j}-\mu_{i}}{\sqrt{\sigma_{i}^{2}+\epsilon }}\times \gamma_{j}+\beta_{j}, \end{array}$$

where $\mu \in R^{N}$ and $\sigma \in R^{N}$ are the mean and standard deviations of x over the channel axis, respectively, and $\gamma \in R^{D}$ and $\beta \in R^{D}$ are trainable scaling and shifting parameters, respectively. ϵ is a small constant used to avoid division by zero and we set $\epsilon =10^{-6}$.

## Appendix C. Multi-Head Attention

We present the details of multi-head attention MHA(·) [14]. Multi-head attention is composed of scaled dot-product attention (SDA) [14], which takes the query $Q\in R^{N_{q}\times D_{q}}$, key $K\in R^{N_{\mathrm{kv}}\times D_{k}}$, and value $V\in R^{N_{\mathrm{kv}}\times D_{v}}$ as the input and sum *V* over the element axis, weighted by the attention computed from the query and key:

(A4) $$\begin{array}{c} \mathrm{SDA}\left(Q,K,V\right)=\mathrm{softmax}\left(\frac{QK^{T}}{\sqrt{D_{k}}}\right)V. \end{array}$$

Let us denote $q\in R^{N\times D}$, $k\in R^{N\times D}$ and $v\in R^{N\times D}$ as token sequences corresponding to the query, key, and value, respectively. In multi-head attention, they are linearly projected by *H* sets of trainable weights and independently fed into SDAs, and the *H* outputs are concatenated and linearly projected:

(A5) $$\begin{array}{c} \mathrm{MHA}\left(q,k,v\right)=\mathrm{Concat}\left[o^{1},o^{2},\ldots ,o^{H}\right]W_{\mathrm{MHA}}, \end{array}$$

(A6) $$\begin{array}{c} o^{h}=\mathrm{SDA}\left(qW_{q}^{h},kW_{k}^{h},vW_{v}^{h}\right), \end{array}$$

where $W_{q}^{h}\in R^{D\times D_{\mathrm{head}}}$, $W_{k}^{h}\in R^{D\times D_{\mathrm{head}}}$, $W_{v}^{h}\in R^{D\times D_{\mathrm{head}}}$, and $W_{\mathrm{MHA}}\in R^{H\cdot D_{\mathrm{head}}\times D}$ are trainable 2D matrices for the linear projections. $D_{\mathrm{head}}$ is typically set to D/H.
